# [Retracted] Long non‑coding RNA NEAT1 promotes the malignancy of laryngeal squamous cell carcinoma by regulating the microRNA‑204‑5p/SEMA4B axis

**DOI:** 10.3892/ol.2023.14148

**Published:** 2023-11-13

**Authors:** Ling Han, Chaopan Zheng, Shihai Wu

Oncol Lett 22: 802, 2021; DOI: 10.3892/ol.2021.13063

Following the publication of this paper, it was drawn to the Editor's attention by a concerned reader that certain of the Transwell cell invasion assay data shown in Fig. 2E on p. 8 were strikingly similar to data that had appeared in different form in a different article by different authors at different research institutes that had already been accepted for publication [Yan C, Gao L, Qiu X and Deng C: Schisandrin B synergizes docetaxel-induced restriction of growth and invasion of cervical cancer cells *in vitro* and *in vivo*. Ann Transl Med 8: 1157, 2020].

Owing to the fact that some of the data in the above article were already under consideration for publication prior to its submission to *Oncology Letters*, the Editor has decided that this paper should be retracted from the Journal. The authors were asked for an explanation to account for these concerns, but the Editorial Office did not receive a satisfactory reply. The Editor apologizes to the readership for any inconvenience caused.

